# Human ALKBH4 Interacts with Proteins Associated with Transcription

**DOI:** 10.1371/journal.pone.0049045

**Published:** 2012-11-08

**Authors:** Linn G. Bjørnstad, Trine J. Meza, Marit Otterlei, Solveig M. Olafsrud, Leonardo A. Meza-Zepeda, Pål Ø. Falnes

**Affiliations:** 1 Department of Molecular Biosciences, University of Oslo, Oslo, Norway; 2 Department of Cancer Research and Molecular Medicine, Norwegian University of Science and Technology, Trondheim, Norway; 3 Genomics Core Facility, Department of Molecular Biosciences, University of Oslo, Oslo, Norway; 4 Department of Tumor Biology, the Norwegian Radium Hospital, Oslo University Hospital, Oslo, Norway; Chinese University of Hong Kong, Hong Kong

## Abstract

The Fe(II)- and 2-oxoglutarate (2OG)-dependent dioxygenase AlkB from *E. coli* is a demethylase which repairs alkyl lesions in DNA, as well as RNA, through a direct reversal mechanism. Humans possess nine AlkB homologs (ALKBH1-8 and FTO). ALKBH2 and ALKBH3 display demethylase activities corresponding to that of AlkB, and both ALKBH8 and FTO are RNA modification enzymes. The biochemical functions of the rest of the homologs are still unknown. To increase our knowledge on the functions of ALKBH4 and ALKBH7 we have here performed yeast two-hybrid screens to identify interaction partners of the two proteins. While no high-confidence hits were detected in the case of ALKBH7, several proteins associated with chromatin and/or involved in transcription were found to interact with ALKBH4. For all interaction partners, the regions mediating binding to ALKBH4 comprised domains previously reported to be involved in interaction with DNA or chromatin. Furthermore, some of these partners showed nuclear co-localization with ALKBH4. However, the global gene expression pattern was only marginally altered upon ALKBH4 over-expression, and larger effects were observed in the case of ALKBH7. Although the molecular function of both proteins remains to be revealed, our findings suggest a role for ALKBH4 in regulation of gene expression or chromatin state.

## Introduction

The superfamily of Fe(II)- and 2-oxoglutarate (2OG)-dependent dioxygenases comprise enzymes which catalyze oxidation reactions in a diverse set of biological processes such as post-translational modification of collagen, the hypoxic response pathway and epigenetic regulation [1]–[6]. These proteins are characterized by their catalytic requirement for ferrous iron as well as the co-substrate 2OG. The primary oxidation reactions they catalyze are coupled to decarboxylation of 2OG, yielding succinate and CO_2_. The *E. coli* AlkB protein [7] is an Fe(II)/2OG dioxygenase involved in DNA and RNA repair, and is induced as part of the adaptive response to alkylation damage. Targeting alkyl lesions at N1-position in purines and N3-position in pyrimidines, AlkB directly reverses the base damage by an oxidative mechanism that involves hydroxylation of the alkyl group, which is consequently destabilized and spontaneously released [8], [9]. The repertoire of AlkB substrates has extended from the originally identified simple methyl lesions 1-methyladenine (1-meA) and 3-methylcytosine (1-meC), to also comprise larger adducts, such as ethyl, propyl and etheno groups [10]–[13], as well as methylated RNA [14].

In mammals, nine AlkB homologs have been reported; ALKBH1-8, as well as the fat mass and obesity protein (FTO) [15], [16]. ALKBH2 and ALKBH3 have similar activities as AlkB, ALKBH2 being most active on dsDNA, while ALKBH3 preferentially demethylates ssDNA and ssRNA [14]. FTO has also, with its weak activity towards 3-methylthymine (3-meT) in ssDNA and 3-methyluracil (3-meU) in ssRNA, been implicated in nucleic acid repair [16], [17], but the recent identification of *N* ^6^-methyladenosine (6-meA) in ssRNA as a preferred substrate indicates a role for FTO in regulating mRNA modification [18]. With the recent demonstration of ALKBH8 being involved in hypermodification of tRNA wobble uridines [19]–[22], the function of the mammalian ALKBH proteins was definitely shown to extend beyond nucleic acid repair. ALKBH1, the mammalian homolog with highest similarity to AlkB, has also been reported to display DNA repair activity [23]. However, the significance of this activity, which has not been confirmed by others, is unclear, and there are indications rather pointing towards a function for ALKBH1 in epigenetic gene regulation, potentially through histone demethylation [24], [25], thereby supporting the suggestion that some of the human ALKBHs are involved in protein demethylation [26], [27]. In line with this, the *S. pombe* AlkB homolog Ofd2 was recently reported to interact with histones [28]. While 2-oxoglutarate decarboxylase activity has been demonstrated for ALKBH4 and ALKBH5 [29], [30], their primary substrates and biological functions still remain, together with those of ALKBH1, ALKBH6 and ALKBH7, to be revealed.

The present study focuses on the human ALKBH4 and ALKBH7 proteins. Through yeast two-hybrid screening, we show binding of ALKBH4 to several proteins with associations to chromatin regulation and transcription. Furthermore, for a selection of these, we demonstrate nuclear co-localization with ALKBH4. However, as revealed through gene expression profiling, ALKBH4 over-expression marginally affects the expression pattern in HEK293 cells, while over-expression of ALKBH7 influences biological pathways such as cell cycle, DNA repair and spermatogenesis, and positively regulates a number of genes involved in meiotic recombination. Thus, this work provides novel insight into the biological function of mammalian AlkB homologs for which no biochemical activity has yet been reported.

## Results

### Yeast Two-hybrid Screens Identified ALKBH4 Binding Partners Involved in Transcription

To improve our knowledge on ALKBH4 and ALKBH7 function, we searched to identify interaction partners of these proteins through yeast two-hybrid (Y2H) screens. Screens were performed using ALKBH4 as bait against two different human libraries, one from placenta and another from fetal brain (Hybrigenics, France). ALKBH7 was used as bait to screen the fetal brain library. While we did not obtain any hits considered highly confident in the ALKBH7 screen, a total of ten such hits were detected in the two screens concerning ALKBH4 (Table 1). Notably, five of these proteins have been associated with transcription and chromatin modification, suggesting a function of ALKBH4 in gene regulation. Of these, the transcriptional co-activator and histone acetyltransferase (HAT) p300 [31] was the only protein identified in both screens and, of note, the highly similar p300 paralog CBP was not detected in any of them. The homeotic transcription factor ATBF1 [32], [33] and the tissue specific heat-shock transcription factor HSF4 [34], [35] were identified as ALKBH4 partners exclusively in the placenta screen. The proteins AF9 and ENL, which have similar biological functions and display very high sequence homology (56% identity throughout the entire sequence) [36] were identified in the placenta and brain screens, respectively. The specific functions of AF9 and ENL are currently unknown, but they have both been associated with histone modification and transcriptional elongation [37]–[39].

**Table 1:** High confidence^a^ ALKBH4 interacting proteins identified by the yeast two-hybrid system.

Cellular process	Gene	Length (aa)	SID^b^	Domain(s)encompassed by SID	Screen		
					Fetal brain	Placenta	Placenta (ALKBH4^H169A/D171A^)
Transcription	*ATBF1*	3703	1545–1626	C_2_H_2_ zinc fingers	−	+	+
	*AF9*	568	10–151	YEATS	+	−	−
	*ENL*	559	4–151	YEATS	−	+	(+)^c^
	*HSF4*	462	1–93	DBD	−	+	+
	*p300*	2414	1054–1352	Bromodomain, PHD	+	+	+
Other	*TES*	421	1–375	PET, LIM1–2	−	+	+
	*EIF3C*	914	749–882	PCI	+^d^	+	−
	*MTMR6*	621	251–539	PTP	+	(+)^c^	−
	*PSMA6*	246	11–232	Proteasome	+	−	−
	*GID: 13396337*	242	13–66	Transmembrane domain	+	−	−

a PBS (predicted biological score) of A or B. PBS (calculated according to [75]) represents the probability of an interaction to be non-specific, and refers to an e-value with defined thresholds to rank the results in the high-to-low confidence categories A–D.

b SID (selected interaction domain) refers to the amino acid region shared by all prey fragments matching the same reference protein,

c (+) Low confidence hit (PBS D),

d EIF3CL. Grey shade, transcription related interactants. YEATS, Yaf9 ENL AF9 TAF14 Sas5; DBD, DNA binding domain; PHD, plant homeodomain; PET, Prickle Espinas Testin; LIM, Lin-11 Isl-1 Mec-3; PCI, Proteasome, COP9, Initiation factor-3; PTP, protein tyrosine phosphatase.

To further investigate whether the observed interactions are dependent on the enzymatic activity of ALKBH4, an enzymatically inactive mutant (ALKBH4^H169A/D171A^) was used as bait in a Y2H screen against the placenta library. Notably, this screen retrieved a very similar set of proteins as the screen performed with wild-type ALKBH4 (Table 1), indicating that the observed interactions can occur independently of the oxygenase activity of ALKBH4.

### ALKBH4 Binding is Mediated by Chromatin-associated Domains

**Figure 1:**
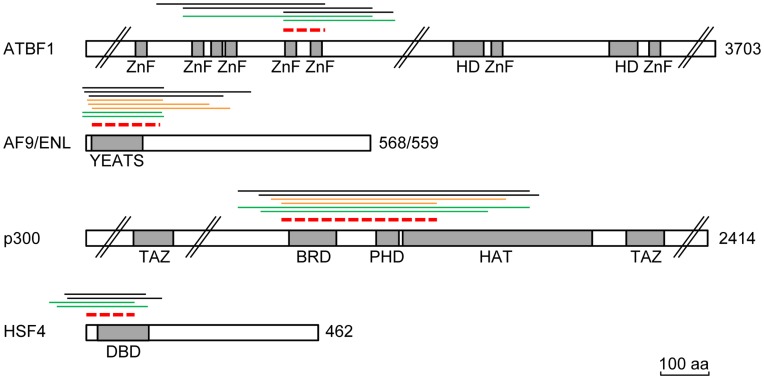
Schematic representation of yeast two-hybrid high confidence hits involved in transcription. Individual prey fragment clones and the resulting selected interaction domains (SIDs) reported to bind ALKBH4 are indicated above each protein; black lines, placenta library; orange lines, fetal brain library; green lines, placenta library screened with ALKBH4^H169A/D171A^; red dashed lines, SIDs. Grey boxes indicate protein domains. Proteins and domains are drawn to scale according to the InterPro (version 4.8) and PROSITE (release 20.68) databases [73], [74].//indicates regions omitted for simplicity. ZnF, C_2_H_2_ zinc finger; HD, homeodomain; YEATS, Yaf9 ENL AF9 Taf14 Sas5; TAZ, transcription adaptor putative zinc finger; BRD, bromodomain; PHD plant homeodomain; HAT histone acetyl transferase; DBD, DNA binding domain. Bar, 100 aa.

The clones identified in the Y2H screens usually represented non-full-length fragments of the interactants. The overlapping sequences present in all clones representing the same partner protein define the part of the protein that is responsible for the interaction, the so-called selected interaction domain (SID). A schematic representation of the transcription/chromatin-related high confidence hits, depicting the SID involved in the interaction with ALKBH4, is shown in Figure 1. Interestingly, for all five proteins the SID encompassed only a limited region, and in all cases this region included annotated domains reported to interact with DNA and/or chromatin. For both AF9 and ENL, the ALKBH4-interacting part mapped to the chromatin-associated YEATS domain, while the portion of ATBF1 that binds ALKBH4 was shown to encompass two of the numerous C_2_H_2_-type zinc fingers found in this protein. Moreover, we found the interaction between ALKBH4 and HSF4 to be mediated through the amino-terminal DNA binding domain (DBD) of the latter. Finally, the region of p300 mediating the ALKBH4-interaction covered both the bromodomain and plant homeodomain (PHD).

Since this region is adjacent to the HAT domain and since bromo domain are known to bind to acetylated proteins, we considered the possibility that ALKBH4 is acetylated by p300. To address this issue, we used an anti-FLAG antibody to immunoprecipitate FLAG-ALKBH4 from cell extracts containing over-expressed p300 as well as FLAG-ALKBH4. However, no *in vivo* ALKBH4 acetylation was detected (data not shown).

### ALKBH4 Co-localizes with the Transcriptional Proteins AF9, ENL and p300 in the Nucleoplasm and Nucleoli

**Figure 2:**
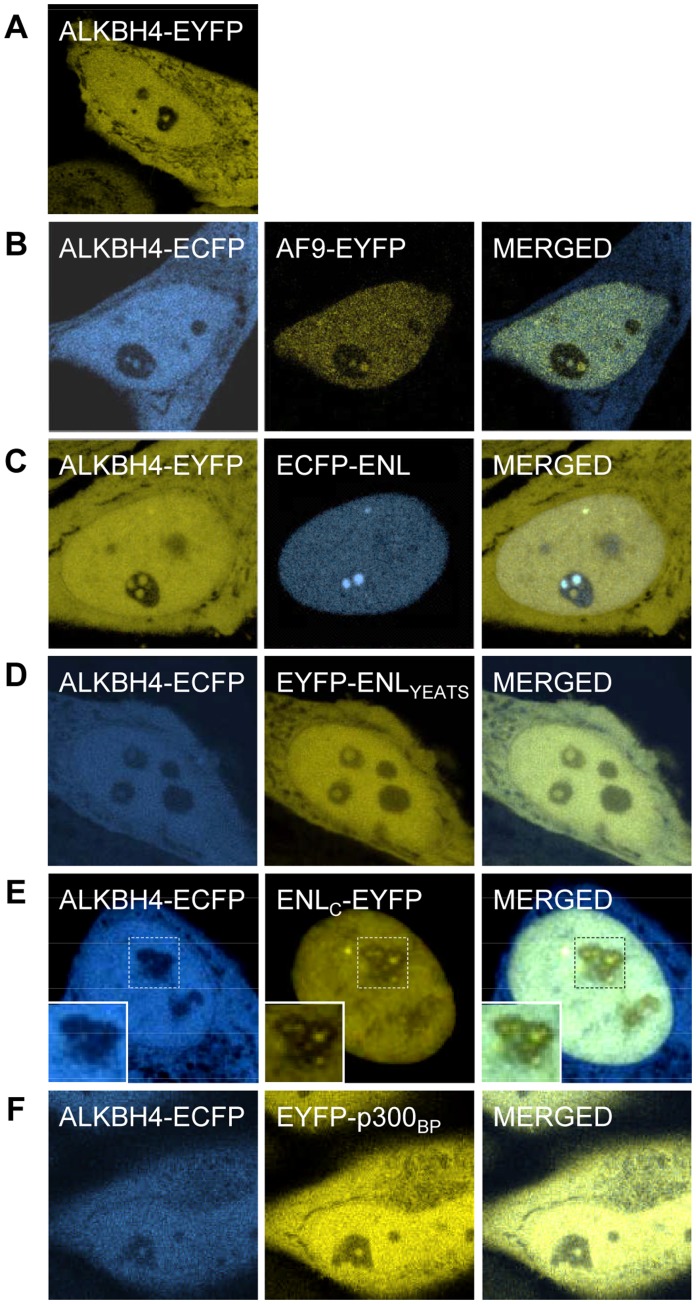
Subcellular localization of ALKBH4 and its co-localization with transcription-associated proteins. (**A**) Subcellular localization of ALKBH4-EYFP in HeLa cells. Co-expression of (**B**) ALKBH4-ECFP and AF9-EYFP, (**C**) ALKBH4-EYFP and ECFP-ENL, (**D**) ALKBH4-ECFP and EYFP-ENL_YEATS_, (**E**) ALKBH4-ECFP and ENL_C_-EYFP and (**F**) ALKBH4-ECFP and EYFP-p300_BP_, as analyzed by confocal fluorescence microscopy. Insets are enlargements of boxed areas.

We have made extensive efforts to verify the interactions that were observed in the Y2H screen by independent methods, such as pull-down experiments with recombinant GST-tagged proteins and co-immunoprecipitation of tagged, co-expressed proteins from cell lysates (experimental outlines can be found in the Materials and Methods section). However, none of these efforts were successful with respect to robust verification, possibly because the interactions are transient and of low affinity. Nonetheless, given the striking overrepresentation of DNA/chromatin binding moieties of transcription-associated proteins among the interactions, we still considered it likely that the corresponding interactions are biologically relevant and occurring in mammalian cells *in vivo*. To investigate this, the chromatin-related proteins that were detected in both screens, AF9, ENL and p300, were selected for co-localization studies with ALKBH4. HeLa cells were transiently co-transfected with a plasmid encoding ALKBH4 fused to either Enhanced Cyan Fluorescent Protein (ALKBH4-ECFP) or Enhanced Yellow Fluorescent Protein (ALKBH4-EYFP) in combination with plasmids encoding EYFP/ECFP-fusions of the selected partner proteins or truncations of these, and localization patterns were analyzed by confocal microscopy. First, we determined the subcellular localization of ALKBH4 (ALKBH4-EYFP) alone, which, consistent with a previous report [40], was found to be localized both in the nucleus and the cytoplasm, with similarly strong signals detected in the two compartments (Figure 2A).

#### Co-localization with the AF9/ENL YEATS domain

ALKBH4 showed nuclear co-localization with both AF9 and ENL in which partial overlap in distinct spots in nucleoplasm and nucleoli was observed for both proteins (Figure 2B and 2C and data not shown). In order to address the significance of the YEATS domain for these presumable interactions, we examined the co-localization patterns of ALKBH4 with two truncated ENL versions, which expressed either the YEATS domain (EYFP-ENL_YEATS_) or the C-terminal part (ENL_C_-EYFP). A co-localization pattern similar to that of full-length ENL was obtained for the YEATS only version (Figure 2D). However, deletion of the YEATS domain strongly reduced the co-localization of ENL with ALKBH4 in nucleoli (Figure 2E). This supports the results from the Y2H screens, strengthening the indication of an interaction between ALKBH4 and AF9/ENL, with the YEATS domain of latter being important for mediating the interaction.

#### Co-localization with the bromodomain and PHD finger of p300

In our Y2H screens, the p300 clones identified to bind ALKBH4 all encompassed both the bromodomain and PHD finger, a protein domain combination that has been shown to compose a functional protein moiety [41]. Thus, in order to determine if p300 co-localizes with ALKBH4, we decided to use an EYFP-tagged fragment of p300 covering the region of the bromodomain and PHD only (aa 1039–1285, EYFP-p300_BP_), instead of the full-length protein. Indeed, similar to what was observed for AF9 and ENL, p300_BP_ co-localized with ALKBH4 in spots in nucleoli (Figure 2F). A similar nuclear localization pattern and nucleolar presence has been previously reported for over-expressed, full-length p300 [42]. Our results thus indicate an interaction between ALKBH4 and p300_BP_. However, the individual contribution of the two domains considered here can not be determined from this experiment.

### ALKBH4 Partly Co-localizes with the RNA Polymerase I Complex

Being the site of ribosome biogenesis, the nucleolus also comprises the process of ribosomal RNA (rRNA) synthesis. Our observations of ALKBH4 co-localizing in nucleolar foci with all three transcription-associated proteins examined made us speculate if ALKBH4 could potentially have a function in transcription of ribosomal DNA (rDNA) genes. We therefore examined whether ALKBH4 and ENL co-localize with the RNA polymerase I subunit RPA43. Thus, HeLa cells were transiently co-transfected with plasmids encoding ALKBH4-EYFP, ECFP-ENL and a Red Fluorescent Protein (RFP)-fusion of RPA43 (RPA43-RFP). RPA43 was observed at the surroundings of the distinct nucleolar ALKBH4/ENL foci with partially overlapping staining observed (Figure S1, insert and nucleolus at the lower right). Partial co-localization of ALKBH4 with ENL in nucleoplasmic and nucleolar spots was detected, as mentioned above. Notably, we observed a larger amount of ENL foci overlapping with RPA43 foci compared to the overlap between ALKBH4 and RPA43. Hence, these results suggest that ALKBH4 is not primarily involved in nucleolar RNA polymerase I-dependent rDNA transcription.

### Effects of ALKBH4 and ALKBH7 on Global Gene Expression and DNA Methylation Patterns

**Figure 3:**
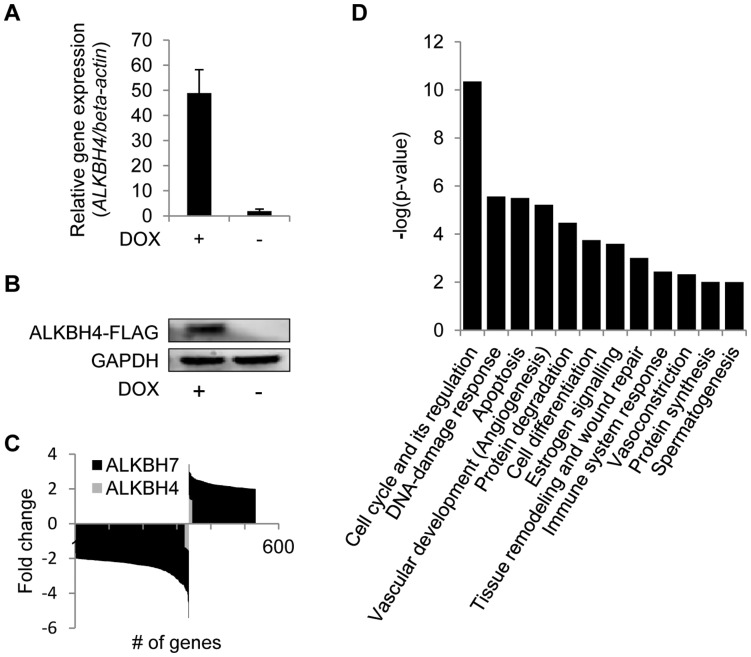
Effects of ectopic expression of ALKBH4 or ALKBH7 on global gene expression. (**A**) Quantitative RT-PCR analysis of relative *ALKBH4* levels in HEK293 cells stably transfected with a construct for DOX-inducible over-expression of ALKBH4-FLAG, either treated with DOX (2 µg/ml) or untreated. Results are presented as mean fold change of three independent replicates normalized to *β-actin* ± S.D. (**B**) Ectopic ALKBH4 protein levels in DOX-induced and non-induced cells, as determined by Western blot analysis. Ectopic ALKBH4 was detected using an antibody against the FLAG-tag introduced at the C-terminus of ALKBH4. GAPDH expression levels are included as loading control. (**C**) Microarray analysis of gene expression in cells over-expressing either ALKBH4 or ALKBH7 vs. non-overexpressing cells. The number of genes whose expression is altered at least 2.0-fold (ALKBH7) or 1.35-fold (ALKBH4) is indicated (**D**) MetaCore (GeneGo Inc.) analysis of molecular pathways significantly (False discovery rate (FDR) <0.05) enriched with genes affected by ectopic ALKBH7 expression. DOX, doxycycline.

We further considered the possibility that ALKBH4 may itself be capable of modulating transcription. To investigate this, we generated a stable HEK293 cell line in which the ALKBH4 encoding gene was introduced at a specific, transcriptionally active, genomic locus, behind a tetracycline-inducible promoter, thereby enabling controlled over-expression of ALKBH4. Ectopic ALKBH4 expression was induced upon treatment with the tetracycline analog doxycycline (DOX), and the resulting ALKBH4 increase was verified on the mRNA and protein levels, by qPCR and Western blotting, respectively (Figures 3A and B). The global gene expression profiles of the ALKBH4 over-expressing cell line and the non-induced, parental cell line were subsequently compared in a microarray-based genome-wide expression analysis. Surprisingly, very small effects on gene expression were observed upon over-expression of ALKBH4. Actually, none of the genes were up- or down-regulated above 2-fold, and only 22 genes showed differential expression when the fold change stringency was reduced to 1.35 (Figure 3C). A list of differentially expressed genes (q-value <5, fold change (FC) >1.35) upon ALKBH4 over-expression is shown in Table 2. Noteworthy, the absence of *ALKBH4* itself among the up-regulated genes in the over-expressing cell line is explained by annealing of the probe to the untranslated region (UTR) of the gene (data not shown). The low number of genes affected by ALKBH4 over-expression indicates that ALKBH4 does not affect transcription at the global level in the HEK293 cells.

**Table 2:** Differentially expressed genes (q-value <5, fold change >1.35) identified in ALKBH4 over-expressing cells compared to parental non over-expressing cells.

	Gene	Description	Fold Change
**Up-regulated** 1	*HSPA1B*	Heat shock 70kDa protein 1B	1.63
	*INSIG1*	Insulin induced gene 1, transcript variant 2	1.43
	*LAMA5*	Laminin, alpha 5	1.43
	*FAM38A*	Family with sequence similarity 38, member A	1.39
	*HSPA8*	Heat shock 70kDa protein 8, transcript variant 1	1.38
	*LOC642031*	Hypothetical protein LOC642031	1.38
	*FASN*	Fatty acid synthase	1.36
	*LOC23117*	KIAA0220-like protein, transcript variant 16	1.35
	*INTS1*	Integrator complex subunit 1	1.35
	*SEC16A*	SEC16 homolog A	1.35
**Down-regulated**	*DDIT4*	DNA-damage-inducible transcript 4	−1.55
	*LGALS1*	Lectin, galactoside-binding, soluble, 1	−1.50
	*LETMD1*	LETM1 domain containing 1, transcript variant 1	−1.48
	*LOC653994*	Similar to Eukaryotic translation initiation factor 4H, transcript variant 2	−1.48
	*SLC3A2*	Solute carrier family 3, member 2, transcript variant 6	−1.45
	*CTH*	Cystathionase (cystathionine gamma-lyase), transcript variant 1	−1.43
	*TSC22D3*	TSC22 domain family, member 3, transcript variant 2	−1.43
	*MCM7*	Minichromosome maintenance complex component 7, transcript variant 1	−1.38
	*DDIT3*	DNA-damage-inducible transcript 3	−1.37
	*STC2*	Stanniocalcin 2	−1.37
	*SNX5*	Sorting nexin 5, transcript variant 1	−1.37
	*RASSF1*	Ras association (RalGDS/AF-6) domain family member 1, transcriptvariant C	−1.36

1 The *ALKBH4* probe was not detected due to annealing to the UTR of the gene. Sorted by fold change.

Furthermore, to characterize the biological functions of the genes affected by ectopic ALKBH4 expression, a gene ontology (GO) analysis was performed using MetaCore GeneGo Pathways Analysis software (GeneGo Inc.). However, the differentially expressed genes were not enriched in any molecular pathway.

Several mammalian AlkB homologs have been shown to be involved in DNA/RNA transactions, and these are basic proteins with a high pI value, thus allowing binding to the negatively charged nucleic acid backbone. In contrast, ALKBH1, ALKBH4 and ALKBH7 are acidic proteins, and it has been proposed that they may be involved in demethylating histones or other proteins (Sedgwick *et al.* 2007). Indeed, genetic ablation of ALKBH1 has been shown to dysregulate a number of genes in the mouse placenta [24], and we were therefore also interested in investigating the effects of ALKBH7 on gene regulation. Using the same approach as for ALKBH4, we generated a stable HEK293 cell line with tetracycline-inducible over-expression of ALKBH7, which was used to determine the effects of ectopic ALKBH7 levels on gene expression. Considerably stronger effects were detected in the case of ALKBH7, compared to ALKBH4. A total of 532 genes, excluding *ALKBH7*, were differentially expressed (q-value <5, FC >2) between the parental and ALKBH7 over-expressing cell lines. Of these, 197 genes were up-regulated and 335 genes were down-regulated (Figure 3C). Notably, ALKBH7 over-expression resulted in a higher number of down-regulated compared to up-regulated genes and, moreover, the most down-regulated genes were more strongly affected (3.8< FC <5.4) than the most up-regulated ones (2.9< FC <3.4). The most up- or down-regulated genes are listed in Table 3.

In order to determine possible enrichment of ALKBH7 affected genes in certain biological pathways, we further performed a GO analysis similar to that of ALKBH4. Twelve GO categories were determined to be significantly overrepresented (false discovery rate (FDR) <0.05) among the differentially expressed genes (Figure 3D). The pathway mostly enriched with ALKBH7 affected genes was “cell cycle and its regulation”, in which 33 of 444 genes displayed expression level alterations upon ectopic ALKBH7 expression. While the great majority of these (31 genes) showed decreased expression, only two genes, *RBX1 (RING-box protein 1)* and *PRKAR1A (PKA-regulatory subunit 1A)*, were up-regulated.

ALKBH7 is annotated in the NCBI database as spermatogenesis-associated protein 11 (*SPATA11*) or spermatogenesis cell proliferation-related protein. Interestingly, the results of our GeneGo pathway analysis included “spermatogenesis” as a category of enriched genes (Figure 3D). Herein, three genes (*CREB1, PRKAR1A and GNAS1*) of a total of 22, belonging to the sub-category “transcription – CREM signaling in testis”, were differentially expressed after ALKBH7 over-expression. Moreover, among the top twenty up-regulated genes, three genes involved in the process of meiotic recombination during gametogenesis were identified: *disrupted meiotic cDNA 1 (DMC1)* [43], *decreased sperm survival 1 (DSS1*) [44] and *male-specific lethal 3-like 1 (MSL3L1)* [45]. Thus, our results are consistent with an association of the *ALKBH7* gene with spermatogenesis.

To complement the gene expression data, we also performed microarray-based CpG methylation profiling in cells over-expressing either ALKBH4 or ALKBH7, as well as in the respective non over-expressing cells. In case of both proteins, the methylation pattern remained strikingly constant upon over-expression (r^2^ = 0.9975 and 0.9916 for ALKBH4 and ALKBH7, respectively. Figure S2), thus excluding a role for ALKBH4 and ALKBH7 in regulation of global CpG methylation.

### In vivo H3K79 Methylation Levels are not Affected by Ectopic ALKBH4 Expression

**Figure 4:**
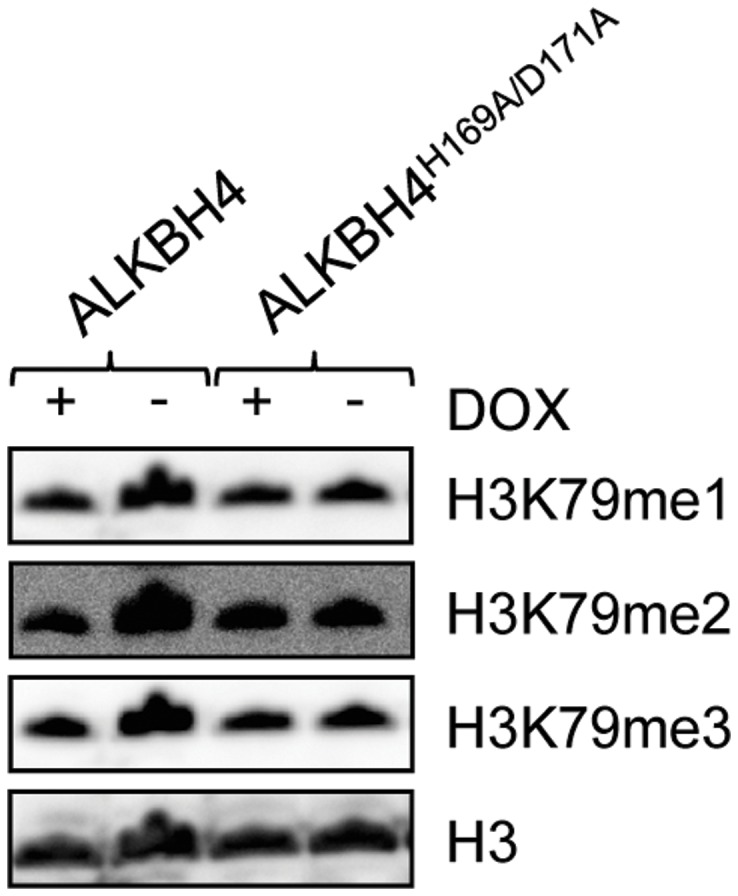
Ectopic ALKBH4 expression does not change H3K79 methylation levels *in vivo*. As analyzed by Western blotting, all three methylation states (mono- di- and tri-methylation) of the H3K79 residue remained similar in histones purified from stable HEK293 transfectants after doxycycline-dependent over-expression of either ALKBH4 or an enzymatically inactive mutant (ALKBH4^H169A/D171A^), compared to the equivalent, non-induced cells. Signal intensities of bands corresponding to methylated histones were quantified using ImageJ [69], and normalized to the total histone H3 load, but no effect of ALKBH4 overexpression was detected (not shown).

Regulation of transcription depends on dynamic chromatin modifications, such as histone methylation, involving proteins with either methyltransferase or demethylase activity. In contrast to the situation for methylated lysine residues found at the flexible histone tails, no demethylase has been found to reverse the activating methyl mark at the lysine 79 (K79) residue in the globular domain of H3, which in mammals is introduced by the enzyme DOT1L. However, there are indications of the existence of such enzyme activity [46], [47], and a study in which treatment of cells with 2-hydroxyglutarate (2HG), an inhibitor of Fe(II)/2OG-dependent oxygenases, resulted in increased H3K79 dimethyl levels has further suggested a member of this protein family to be the enzyme responsible for H3K79 demethylation [48]. Moreover, ALKBH4 has previously been suggested to possess protein demethylase activity [26], and our results imply a role for ALKBH4 in chromatin regulation. Since we found ALKBH4 to interact with the DOT1L-associated proteins AF9 and ENL, we speculated that ALKBH4 could potentially function as a demethylase with specificity for methylated H3K79. In order to address this issue, we isolated histones from the DOX-inducible ALKBH4 over-expressing HEK293 cell line before and after DOX induction, as well as from a similarly generated cell line possessing the ability of stable, inducible over-expression of an enzymatically inactive ALKBH4 mutant (ALKBH4^H169A/D171A^). Subsequently, the methylation status at the H3K79 position in the histones was determined by Western blotting. However, we did not observe any effect of ectopic ALKBH4 expression on the methylation status of histone H3K79, as similar levels of mono-, di- and trimethylated H3K79 were detected in the non-overexpressing cells as well as in those over-expressing ALKBH4^H169A/D171A^ (Figure 4). This suggests that ALKBH4, if involved in demethylation of this histone residue, might be restricted to certain, presently unknown, conditions to be functional.

## Discussion

In the present work, we report that proteins involved in transcription were strongly over-represented among interactants of the human oxygenase ALKBH4 identified by Y2H screens, while no convincing partners were detected for the related ALKBH7 protein. Interestingly, the regions of these transcription-associated interactants that was responsible for interaction with ALKBH4, in all cases encompassed domains which are involved in interaction with DNA and chromatin, and for some of these interactants we observed a co-localization with ALKBH4 in distinct foci in the nucleus. We also performed a global analysis of gene expression and CpG methylation changes induced by ectopic over-expression of ALKBH4 and ALKBH7. While we saw rather small effects of ALKBH4 on both expression and methylation, larger effects on gene expression were observed in the case of ALKBH7.

### Protein Partners Suggest a Role for ALKBH4 in Gene Regulation

Our identification of the transcriptional co-activator p300 as an ALKBH4 partner is indicative of a gene regulatory role of ALKBH4. The region of p300 mediating the ALKBH4-interaction covered both the bromodomain and plant homeodomain (PHD) of this protein. Both domains represent motifs found in chromatin modification effector proteins, thus functioning in recruitment of remodeling-complexes to chromatin. While bromodomains recognize acetylated histones [49], the zinc-coordinating PHD finger is generally involved in binding methylated or unmodified histone H3 [50]. However, PHD fingers are often found close to bromo- or chromodomains [51], and several reports have shown a combinatorial function of the two domains [52], [53]. The function of the p300 PHD finger is still to be elucidated, although it has been reported to be required, together with the bromodomain, for *in vitro* recognition of acetylated nucleosomes [41]. Notably, the histone acetyl transferase CBP, which is highly similar to p300, was not identified as an ALKBH4 interaction partner in the yeast two-hybrid screen. However, p300 HAT activity does not, in contrast to that of CBP, depend on its PHD finger, implying non-redundant functions for these co-activators [54].

Further supporting an involvement of ALKBH4 in gene regulation, we also detected the homologs AF9 and ENL as interacting partners. In addition to a hydrophobic, transcription-associated C-terminus both proteins contain an N-terminal YEATS domain [37], which we here found to mediate the ALKBH4 interaction. The YEATS domain has been named after proteins that carry such a domain (Yaf9, ENL, AF9, Taf14 and Sas5), and many of these proteins are components in transcriptional or chromatin-modifying complexes. The specific function of YEATS has not yet been determined, but several findings indicate a role in chromatin binding. The interaction between ENL and histones H1 and H3 has previously been demonstrated to be mediated through YEATS [55], and recently, AF9/ENL YEATS was found to be the module responsible for recruitment of the super elongation complex (SEC) to chromatin and the elongating Polymerase II [56]. Moreover, as a result of the first three-dimensional YEATS structure, this domain was recently suggested to provide an additional reader module of chromatin, analogous to the bromodomain and PHD finger [57], [58], with the Yaf9 YEATS domain suggested to potentially bind acetylated lysine-residues in histones [57].

The interactions of ALKBH4 with the transcription factors HSF4 and ATBF1 were found to involve domains with the ability of DNA binding, these being the DNA binding domain (DBD) of HSF4 and two of the multiple C_2_H_2_-type zinc fingers of ATBF1. The versatile C_2_H_2_ motif, which is frequently found in gene regulators, has been demonstrated to mediate interactions with proteins as well as both DNA and RNA (reviewed in [59]). Previously, two of the ATBF1 zinc fingers have been reported to bind protein [60].

Interestingly, three of the transcription-associated ALKBH4 partners, AF9, ENL and p300, have all been reported to be fused to the histone methyltransferase MLL in mixed-lineage leukemia (MLL) [37], [61], [62], which is characterized by abberant H3K79 dimethylation profiles [63]. While the ALKBH4 interacting YEATS domain of AF9/ENL is not present in the fusion proteins MLL-AF9 and MLL-ENL [37], the less frequent MLL-p300 fusions, which are thought to promote leukemogenesis through aberrant histone acetylation rather than methylation [61], [64], retain the ALKBH4 interacting p300 region.

**Table 3:** The genes most differentially expressed in either direction identified in ALKBH7 over-expressing cells compared to parental non over-expressing cells.

	Gene	Description	Fold Change
**Up-regulated**	*ALKBH7*	AlkB, alkylation repair homolog 7	13.54
	*MSL3L1*	Male-specific lethal 3-like 1, transcript variant 1	3.41
	*LOC643287*	Similar to prothymosin alpha, transcript variant 1 (LOC643287)	2.97
	*RPL21*	Ribosomal protein L21	2.96
	*NBPF20*	Neuroblastoma breakpoint family, member 20	2.95
	*PEBP1*	Phosphatidylethanolamine binding protein 1	2.90
	*GNL3L*	Guanine nucleotide binding protein-like 3 (nucleolar)-like	2.89
	*SHFM1*	Split hand/foot malformation (ectrodactyly) type 1	2.85
**Down-regulated**	*LOC650215*	Similar to Exportin-T (tRNA exportin)	-5.42
	*PKMYT1*	Protein kinase, membrane associated tyrosine/threonine 1, transcript variant 2	-4.53
	*GDF15*	Growth differentiation factor 15	-4.43
	*LOC643031*	Similar to NADH dehydrogenase subunit 5	-4.06
	*MAZ*	MYC-associated zinc finger protein, transcript variant 2	-3.93
	*CCT7*	Chaperonin containing TCP1, subunit 7 (eta), transcript variant 1	-3.91
	*NGRN*	Neugrin, neurite outgrowth associated (NGRN), transcript variant 1	-3.87
	*LOC727761*	Similar to Deoxythymidylate kinase, transcript variant 4	-3.86
	*CNBP*	CCHC-type zinc finger, nucleic acid binding protein	-3.85
	*MORF4L2*	Mortality factor 4 like 2	-3.80

### Molecular Function of ALKBH4

Although the present work suggests that ALKBH4 may be involved in processes such as chromatin regulation and transcription, its molecular function remains an enigma. Interestingly, two of the herein identified ALKBH4 interacting proteins with associations to chromatin regulation and transcription, AF9 and ENL, also interact with DOT1L, the enzyme responsible for methylation of lysine 79 at histone H3 (H3K79). While no demethylase with specificity for this residue has been reported so far, there are indications of such activity [46], [47]. The responsible enzyme has been suggested to be an Fe(II)/2OG dioxygenase, as elevated H3K79 dimethyl levels has been observed as a result of Fe(II)/2OG dioxygenase inhibition [48]. Intriguingly, plants lack both H3K79 methylation and DOT1L orthologs [65], as well as ALKBH4. Thus, ALKBH4 could be imagined to function as an H3K79 demethylase. Consequently, we investigated whether ectopic ALKBH4 expression affected the cellular H3K79 methylation status in HEK293 cells. However, we did not detect alterations in the levels of neither mono-, di- nor trimethylated H3K79 after ALKBH4 over-expression. Related Fe(II)/2OG dioxygenase activities include JumonjiC (JmjC) protein mediated histone demethylation [2], structural and gene regulatory protein hydroxylation by prolyl-4-hydroxylase (P4H) and prolyl hydroxylase domain containing (PHD) 1–3, respectively [5], [66], as well as epigenetic DNA hydroxylation by the TET enzyme family, which was recently reported to convert 5-methylcytosine (5-meC) to 5-hydroxymethylcytosine (5-hmC) [1]. Previously, the low pI-value of ALKBH4 has suggested this enzyme to possess a protein, rather than a nucleic acid substrate [26], and, consistently, no activity towards the classical AlkB substrates 1-meA and 3-meC has been detected [67]. Furthermore, we previously showed uncoupled decarboxylase activity for ALKBH4, indicating that this protein is a *bona fide* Fe(II)/2OG dioxygenase [30].Thus, a demethylase or hydroxylase activity targeting proteins or, less likely, nucleic acids, could be envisioned for ALKBH4.

### Association of Genes Affected by ALKBH7 Over-expression with DNA Recombination

The previous annotation of *ALKBH7* being associated with spermatogenesis is consistent with the data presented here, as functional annotation analysis revealed spermatogenesis as one of the biological pathways enriched with genes differentially expressed upon ectopic ALKBH7 expression. Meiotic recombination is one of the central events occurring during spermatogenesis, and, consistently, three genes with functions in meiotic recombination were found among the twenty most up-regulated ones. This, in combination with the over-representation of genes related to cell cycle regulation and the DNA damage response among the differentially expressed genes, may indicate a possible role for ALKBH7 in the process of homologous recombination repair (HRR) of double strand breaks (DSBs) introduced by DNA damage as well as in homologous recombination (HR) during meiosis, and maybe also mitosis. Thus, we speculate whether ALKBH7 might function in general regulation of cellular responses to repair of DNA double strand breaks by homologous recombination.

### Conclusions

In the current work we have identified several partners of the ALKBH4 protein and an analysis of these partners suggests some processes, such as chromatin regulation and transcription, in which ALKBH4 may play an important part. Moreover, through analysis of global gene expression changes in response to ALKBH7 over-expression, we observed enrichment of differentially expressed genes in processes like cell cycle and spermatogenesis, supporting the previous annotation of ALKBH7 as a cell proliferation and spermatogenesis-related protein. Obviously, more rigorous studies are required to firmly establish the possible role of ALKBH4 and ALKBH7 in these processes, but the present study may represent a useful starting point.

## Materials and Methods

### Yeast Two-hybrid Assays

Two libraries (Human Placenta RP4 and Human Fetal Brain RP1) were screened using full-length human ALKBH4 as bait (N-LexA-ALKBH4-C fusion). Additionally, an enzymatically inactive ALKBH4 mutant (ALKBH4^H169A/D171A^; mutagenesis described in [30]) was used as bait to screen the human placenta RP4 library. All screens and subsequent data analysis were performed by Hybrigenics.

### Plasmid Construction

For generation of ALKBH4-ECFP and -EYFP fusion constructs, human ALKBH4 cDNA was subcloned between the EcoRI and BamHI sites of the pECFP-N1 and pEYFP-N1 vectors (Clontech), respectively. To prepare the AF9-EYFP plasmid, *AF9* (IMAGE-5298142) was PCR amplified (primers *AF9-fwd* and *AF9-rev*, primer sequences can be found in Table S1) and cloned into the XhoI/KpnI site of pEYFP-N1. pSXG-p300BP [41] was used for amplification of the bromodomain and PHD finger encoding fragment of p300 (primers *p300_BP_-fwd* and *p300_BP_-rev*), which was cloned into the XhoI/EcoRI site of pEYFP-C1 (Clontech), generating the EYFP-p300_BP_ plasmid. To ensure nuclear access of the EYFP-p300_BP_ fusion protein, a sequence encoding a nuclear localization signal (NLS) was subsequently PCR amplified from pCMV-nucEGFP-BP-HA (kindly provided by R. Aasland) using primers *NLS-fwd* and *NLS-rev,* and cloned into the EcoRI/KpnI site of the EYFP-p300_BP_ plasmid. The ENL open reading frame was amplified from pBSIISK+ENL (kindly provided by R. Slany), using primers *ENL-fwd* and *ENL-rev*, and cloned into the XhoI/KpnI site of pEYFP-N1 (Clontech), generating the ENL-EYFP construct. A fragment encoding the N-terminus of ENL (aa 1–141) was amplified (primers *ENL-fwd* and *YEATS-rev*) and cloned into the XhoI/KpnI site of pEYFP-C1, resulting in the EYFP-ENL_YEATS_ construct, encoding a YEATS domain fusion. The XhoI/KpnI site was also used for cloning of the ENL_C_-EYFP fusion construct (primers *ENL_C_-fwd* and *ENL-rev*), in which the YEATS domain encoding part was deleted (aa 142–559).

For generation of inducible, stable cell lines expressing either ALKBH4, ALKBH4^H169A/D171A^ or ALKBH7 (described below), the respective cDNAs were amplified (primer sequences can be found in Table S1) and cloned between the BamHI and EcoRV sites of pcDNA5/FRT/TO (Invitrogen), resulting in the plasmids pcDNA5/FRT/TO-ALKBH4, pcDNA5/FRT/TO-ALKBH4^H169A/D171A^ and pcDNA5/FRT/TO-ALKBH7. A FLAG epitope tag was introduced in the reverse primers for simple detection of the proteins. All constructs were verified by DNA sequencing.

### In vivo Acetylation Assay

The assay was performed essentially as previously described in [68]. Briefly, plasmids encoding FLAG-ALKBH4 (pCIneoB-3xFLAG-ALKBH4) and p300 (pCMVβ-p300) were transiently co-transfected into HCT116 cells using FuGene6 (Roche) according to the manufacturer’s specifications. Empty vectors were used as controls. Twenty four hours after transfection, the cells were treated with the deacetylase inhibitor Trichostatin A (TSA, 2 µM) for 30 minutes or left untreated. Total cell extracts were prepared in lysis buffer (50 mM HEPES pH 7.9, 420 mM NaCl, 0.5% NP-40, 1 mM phenylmethylsulfonyl fluoride (PMSF), 0.5 mM dithiothreitol (DTT) and Complete protease inhibitor cocktail (Roche)). Subsequently, 1–2 mg total cell extract and 2–5 µg anti-FLAG antibody (Sigma F3165) was used for immunoprecipitation of FLAG-ALKBH4. The resulting precipitates were analyzed by Western blotting using an anti-acetyl-lysine antibody (Upstate 06-933) followed by reprobing of the membrane with the anti-FLAG antibody.

### GST Pull-down Assay

The TNT T7 Quick for PCR DNA system (Promega) was used according to the manufacturer’s descriptions to produce *in vitro* transcribed/translated [^35^S]-methionine labelled ALKBH4 in presence of Redivue L-[^35^S]-methionine (GE Healthcare). To minimize production of truncated protein products due to partly degraded mRNA, 10–15 µg RibonucleaseA (Sigma) was added to the reaction mixtures subsequent to translation and further incubated at room temperature for 10 minutes. GST fusions of ENL and p300_BP_ were separately expressed in *E. coli*, immobilized on Glutathione Sepharose resin (GE Healthcare) and washed with interaction buffer (50 mM Tris-HCl pH 8.5, 12 mM NaCl, 0.1 mM ZnAc, 150 mM KCl, 2 mM MgCl_2_, 10 mM 2-mercaptoethanol, 0.1% Triton X-100). *In vitro* translation reaction mixture (5–10 µl) was added to the immobilized GST-fusion proteins, which were incubated with gentle agitation for 30 min at room temperature. Reactions were subsequently subjected to four washes with interaction buffer and one wash with 50 mM Tris-HCl pH 8.0 prior to elution with GST-elution buffer (50 mM Tris-HCl pH 8.0, 15 mM reduced glutathione, 0.1 mM ZnAc). Similarly, reciprocal pull-downs using GST-ALKBH4 and [^35^S]-labelled p300_BP_ (alternatively full-length p300) were also performed. GST-only was included as control. Eluted proteins were analyzed by SDS-PAGE followed by phosphor imaging using LE Storage Phosphor Screens (GE Healthcare) and an exposure time of 1–4 days. Signals were visualized in a Typhoon™ 9400 scanner (GE Healthcare).

### Co-immunoprecipitation

Plasmids encoding HA-tagged versions of ENL, AF9 or p300_BP_, or the empty vector (pCMV-script, Stratagene), were separately transfected into Flp-In-293-ALKBH4 cells (described below) using FuGene6 (Roche) according to the manufacturer’s descriptions. Cells were treated with 2 µg/ml DOX to induce expression of FLAG-tagged ALKBH or left untreated. After 18–24 hours, the cells were harvested in phosphate-buffered saline (PBS) and washed once in PBS before 5–6×10^6^ cells were subjected to crosslinking in PBS with 0.125–0.25% formaldehyde for 20 minutes at 37°C. The crosslinking reaction was stopped by the addition of 0.15 M glycine followed by incubation on ice for 3 minutes and subsequently at room temperature for 2 minutes.

To prepare cell extracts, the cells were washed twice with PBS and resuspended in 3 volumes (∼200 µl) of Buffer I (20 mM HEPES pH 7.9, 1.5 mM MgCl_2_, 100 mM KCl, 0.2 mM EDTA, 20% glycerol, 0.5% NP-40, 1 mM DTT, Complete protease inhibitor cocktail (Roche)) containing 2 µl Omnicleave Endonuclease (200 U/µl Epicentre Technologies) followed by sonication, addition of DNase/RNase cocktail (200 U/µl Omnicleave Endonuclease, 250 U/ml Benzonase (Novagen), 30 mg/ml RNase (Sigma-Aldrich), 10 U/µl DNase (Roche), 100–300 U/mg micrococcal nuclease (Sigma-Aldrich)), incubation with gentle agitation for 1 hour at room temperature and subsequently over night at 4°C. The extracts were cleared by centrifugation at 14,000×*g* at 4°C for 10 minutes.

Immunoprecipitation was performed by pre-incubating the cell extracts with 2–5 µg anti-FLAG antibody (Sigma F3165) in 5 ml Buffer II (20 mM HEPES pH 7.9, 1.5 mM MgCl_2_, 100 mM KCl, 0.2 mM EDTA, 10% glycerol, 1 mM DTT, Complete protease inhibitor cocktail (Roche)) at room temperature for 1 hour. Subsequently, 50 µl 10% Sepharose rec-Protein G (Invitrogen) was added and the samples were incubated with gentle agitation at 4°C for 1 hour. After extensive washing with Buffer II the immunocomplexes were resuspended in Laemmli buffer. Crosslinks were reversed by incubating the samples at 70°C for 10 minutes followed by addition of 0.1 M DTT and further incubation at 95°C for 30 minutes. Immunocomplexes were analyzed by SDS-PAGE and Western blotting using an anti-HA antibody (Abcam Ab9110), and membranes were reprobed with the anti-FLAG antibody.

### Confocal Imaging

Live HeLa S3 cells (ATCC) were examined 16–24 h after transient transfection (using FuGene 6 or FuGene HD (Roche) according to the manufacturer’s recommendations) of the ECFP/EYFP/RFP fusion constructs. The fluorescent images were acquired using a Zeiss LSM 510 Meta laser scanning microscope equipped with a Plan-Apochromate 63×/1.4 oil immersion objective. The images were acquired in the growth medium of the cell, with the stage heated to 37°C, using the Zeiss LSM 510 software. ECFP was excited at λ = 458 nm and detected at λ = 470–500 nm and EYFP was excited at λ = 514 nm and detected at λ = 530–600 nm. The thickness of the slice was 1 µm. All images were acquired with consecutive scans to avoid bleed through. No image processing, except contrast and intensity adjustments, were performed.

### Establishment and Maintenance of Stable, Inducible Cell Lines

Inducible cell lines for stable over-expression of FLAG epitope-tagged versions of ALKBH4, ALKBH4^H169/D171A^ and ALKBH7, respectively, were generated using the Flp-In™ T-REx™ System (Invitrogen) and the Flp-In™ T-REx™-293 host cell line (Invitrogen, R780-07), according to the manufacturer’s specifications. This system ensures isogenic cDNA expression from a single transcriptionally active genomic locus. Briefly, pcDNA5/FRT/TO-ALKBH4, pcDNA5/FRT/TO- ALKBH4^H169/D171A^ and pcDNA5/FRT/TO-ALKBH7 (described above) were independently co-transfected with the Flp recombinase-encoding pOGG44 vector into the Flp-In™ T-REx™-293 host cell line, using FuGene6 transfection agent (Roche). Selection of transfectants was performed in presence of 200 µg/ml Hygromycin B (Clontech). Single colonies conferring hygromycin B resistance and zeocin sensitivity were expanded and treated with doxycycline (Clontech). Total cell extracts were prepared using RIPA lysis buffer (Santa Cruz), according to standard methods, and screened for ALKBH over-expression by Western blotting (described below). Stable transfectants were maintained in Dulbecco’s modified Eagle’s medium (DMEM) (Lonza) supplemented with 10% tetracycline-free fetal bovine serum (FBS) (Clontech), 100 U/ml penicillin (Lonza), 100 U/ml streptomycin (Lonza), 2 mM L-glutamine (Lonza), 15 µg/ml blasticidin-S (Invitrogen) and 200 µg/ml hygromycin B. Ectopic ALKBH expression was induced by addition of 2 µg/ml doxycycline (DOX) to the medium. For microarray analysis, transgene expression was induced at a cell confluence level of approximately 50%. Cells were harvested 48 hours after induction. All samples were prepared in triplicates.

### Histone Purification

Histones were purified from Flp-In™-293 cells (Invitrogen) containing a stable, doxycycline-inducible integration expressing FLAG-ALKBH4 or FLAG-ALKBH4^H169A/D171A^ (described above), either treated with doxycycline or untreated, using the Histone purification mini kit (Active Motif) according to the manufacturer’s specifications. Briefly, cells were lysed in Extraction Buffer at 4°C for 1 hour. Cleared lysates were neutralized and loaded onto pre-equilibrated spin columns which were washed prior to histone elution. Purified histones were subsequently concentrated by perchloric acid precipitation.

### Antibodies and Western Blot Analysis

Total cell extracts or histones were subjected to separation by SDS-PAGE (NuPAGE® SDS-PAGE Gel System, Invitrogen) and transferred onto Invitrolon PVDF membranes (Invitrogen). Membranes were subjected to blocking with 5% dry milk in PBS with 0.1% Tween-20 (PBS-T) for 1 hour. After incubation with primary antibody diluted in PBS-T with 5% dry milk for 1 hour, the membranes were washed three times with PBS-T, incubated with secondary antibody diluted in PBS-T with 2,5% dry milk for 1 hour and subjected to three additional washes with PBS-T. Primary antibodies used were anti-FLAG (Sigma F3165), anti-HA (Abcam Ab9110), anti-GAPDH (Applied Biosystems AM4300), anti-acetyl-lysine (Upstate 06-933) anti-H3K79me1 (Abcam ab2886), anti-H3K79me2 (Abcam ab3594), anti-H3K79me3 (Abcam ab2621) and anti-H3 (Abcam ab1791). While the anti-H3K79me1 and anti-H3K79me3 antibodies were specific towards mono- and trimethylated H3K79, respectively, anti-H3K79me2 antibody specificity towards dimethylated H3K79 was ensured by pre-incubating the antibody with two H3 derived peptides containing either mono- (Abcam ab4555) or trimethylated (Abcam ab4557) H3K79 (1 µg/ml of each) for 30 min at RT before incubation with the membrane, thereby blocking mono- and trimethyl reactive sites (data not shown). Anti-FLAG and anti-GAPDH antibodies were used in combination with an alkaline phosphatase-conjugated secondary antibody and the Amersham ECF detection system (GE Healthcare). Fluorescence signal detection was performed in a Typhoon scanner 9400 (GE Healthcare). The remaining antibodies were used in combination with a horseradish peroxidase-conjugated secondary antibody and the SuperSignal West Dura kit (Thermo Scientific). Chemiluminescence signals were visualized on a Kodak Image Station 4000R Pro instrument (Carestream Health). Densitometry was performed using the ImageJ software [69].

### RNA and Genomic DNA Isolation

Total RNA and genomic DNA were isolated from cell lines over-expressing ALKBH4 or ALKBH7 or the equivalent non-induced cell lines. For RNA isolation, TRIzol (Invitrogen) was used according to the manufacturer’s descriptions. The RNA was subsequently subjected to a clean-up step using the RNeasy Mini Kit (Qiagen) according to the RNA Cleanup procedure in the supplied manual. Genomic DNA was isolated using the QIAamp DNA Mini Kit (Qiagen) according to the manufacturer’s protocol.

### Quantitative PCR Analysis

SuperScript II Reverse Transcriptase (Invitrogen) was used to synthesize cDNA, using oligo(dT)_12−18_ primers (Invitrogen) and 5 µg of total RNA. cDNA synthesis was performed according to the manufacturer’s specifications, except that the incubation step prior to addition of the SuperScript II RT was omitted. Quantitative PCR (qPCR) was performed in 20 µl reactions using SYBR Green master mix (Qiagen), 4 µl cDNA (diluted 1∶16), and 10 pmol *ALKBH4*-specific primers (*ALKBH4-qPCR-fwd* and *ALKBH4-qPCR-rev*, primer sequences can be found in Table S1). Reactions were performed on a LightCycler 1.5 instrument (Roche). LightCycler software 3.5.3 (Roche) was used for data analysis and quantification was performed using the ΔC_T_ method [70] with *β-actin* as endogenous reference (primers *beta-actin-fwd* and *beta-actin-rev*). All experiments were performed in triplicates.

### Microarrays and Data Analysis

mRNA expression and CpG methylation profiling was performed by the Helse Sør-Øst/University of Oslo Genomics Core Facility using the Illumina HumanWG-6 v3 Expression BeadChip and Illumina Infinium HumanMethylation27 BeadChip, respectively, according to the manufacturer’s protocols. Data extraction and initial quality control of the bead summary raw data were performed using GenomeStudio v2011.1 from Illumina and the Gene Expression and Methylation module v1.9.0. The data was annotated using the HumanWG-6_V3_0_R3_11282955_A and HumanMethylation27_270596_v1.2 annotation files from Illumina. Unnormalized bead intensities were exported into a tab delimited text file and imported into J-express Pro (v. 2.7) for further downstream analysis [71]. Bead intensities were quantile normalized, and rank product analysis was performed to identify gene expression changes [72]. A significant threshold of q-value <5 and a fold-change larger than 1.35 for ALKBH4 or 2 for ALKBH7 was used to identify differentially expressed genes. Differentially expressed genes were further analyzed in MetaCore (GeneGo) to identify functional enrichment.

CpG methylation data was analyzed using the GenomeStudio Methylation Module, where avgBeta (average ratio of signal from methylated probe relative to the sum of both methylated and unmethylated probes) was calculated and used for comparisons within sample groups.

The mRNA expression and CpG methylation datasets have been deposited in the GEO data repository (www.ncbi.nlm.nih.gov/geo/, accession number GSE39135).

## Supporting Information

Figure S1 **Partial co-localization of ALKBH4 and ENL with RNA Polymerase I subunit RPA43 in nucleolar speckles.** Co-expression of ALKBH4-EYFP with ECFP-ENL and RPA43-RFP in HeLa cells, as analyzed by confocal fluorescence microscopy. Insets are enlargements of boxed areas.
(PDF)

Figure S2 **Effects of ALKBH4 and ALKBH7 over-expression on the global DNA methylation pattern.** CpG methylation profiles were analyzed in stably transfected HEK293 cells before vs. after doxycycline induced over-expression of ALKBH4 (left panel) or ALKBH7 (right panel), using the Illumina Infinium HumanMethylation27 BeadChip. DOX, doxycycline.
(PDF)

Table S1 **Primer sequences.**
(PDF)
