# Supplementary material for: Human ALKBH4 Interacts with Proteins Associated with Transcription
Source: PLoS One. 2012 Nov 8;7(11):e49045. doi: 10.1371/journal.pone.0049045 (PMC3493508; doi:10.1371/journal.pone.0049045)
Supplement: Figure S1 — Partial co-localization of ALKBH4 and ENL with RNA Polymerase I subunit RPA43 in nucleolar speckles. Co-expression of ALKBH4-EYFP with ECFP-ENL and RPA43-RFP in HeLa cells, as analyzed by confocal fluorescence microscopy. Insets are enlargements of boxed areas. (PDF) [file pone.0049045.s001.pdf]

Bjørnstad *et al.* – Supplementary Figure S1

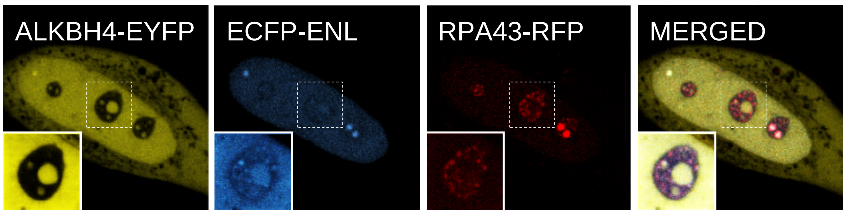

Supplementary Figure S1. Partial co-localization of ALKBH4 and ENL with RNA Polymerase I subunit RPA43 in nucleolar speckles. Co-expression of ALKBH4-EYFP with ECFP-ENL and RPA43-RFP in HeLa cells, as analyzed by confocal fluorescence microscopy. Insets are enlargements of boxed areas.
