# Supplementary material for: Human ALKBH4 Interacts with Proteins Associated with Transcription
Source: PLoS One. 2012 Nov 8;7(11):e49045. doi: 10.1371/journal.pone.0049045 (PMC3493508; doi:10.1371/journal.pone.0049045)
Supplement: Figure S2 — Effects of ALKBH4 and ALKBH7 over-expression on the global DNA methylation pattern. CpG methylation profiles were analyzed in stably transfected HEK293 cells before vs. after doxycycline induced over-expression of ALKBH4 (left panel) or ALKBH7 (right panel), using the Illumina Infinium HumanMethylation27 BeadChip. DOX, doxycycline. (PDF) [file pone.0049045.s002.pdf]

Bjørnstad *et al.* – Supplementary Figure S2

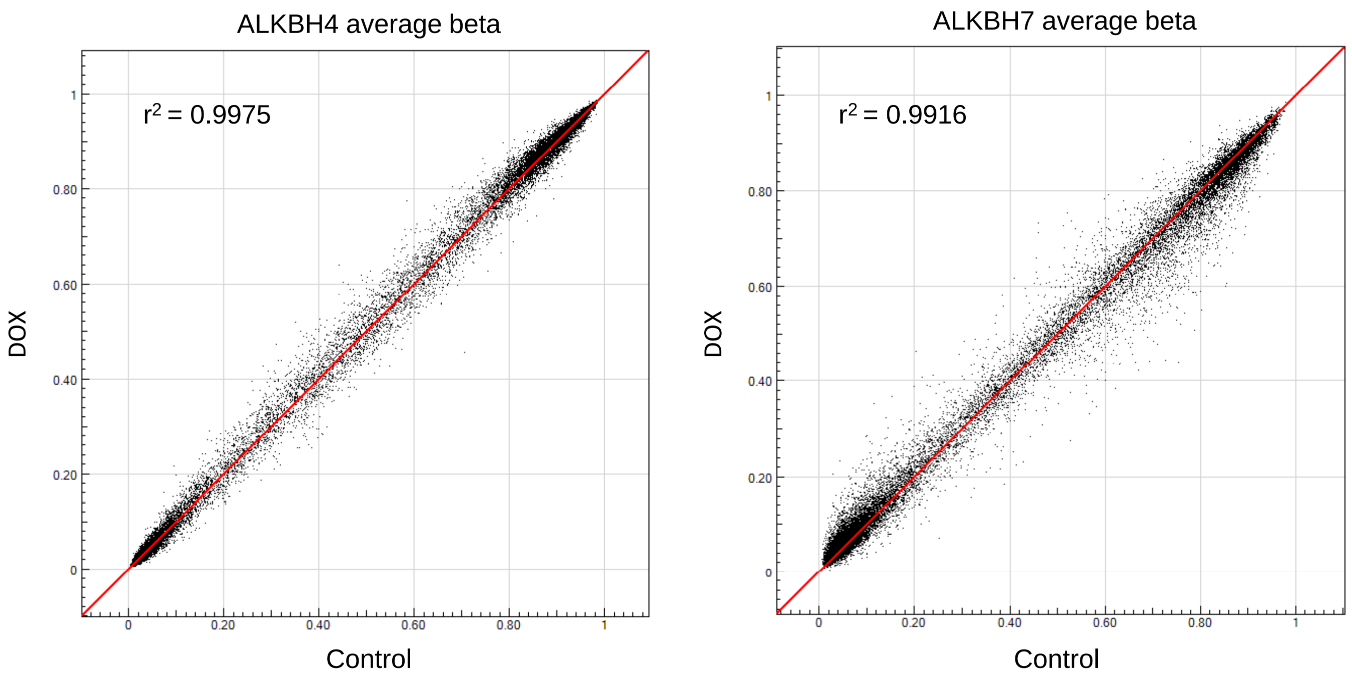

Supplementary Figure S2. Effects of ALKBH4 and ALKBH7 over-expression on the global DNA methylation pattern. CpG methylation profiles were analyzed in stably transfected HEK293 cells before vs. after doxycycline-induced over-expression of ALKBH4 (left panel) or ALKBH7 (right panel), using the Illumina Infinium HumanMethylation27 BeadChip. DOX, doxycycline.
