# Supplementary material for: Human ALKBH4 Interacts with Proteins Associated with Transcription
Source: PLoS One. 2012 Nov 8;7(11):e49045. doi: 10.1371/journal.pone.0049045 (PMC3493508; doi:10.1371/journal.pone.0049045)
Supplement: Table S1 — Primer sequences. (PDF) [file pone.0049045.s003.pdf]

Bjørnstad *et al.*- Supplementary Table S1

Supplementary Table S1. Primer sequences

Primer name	Primer sequence (5'-3')	Application
$p300_{BP}$-fwd	ATCGAGTACTCGAGTCATGGCCCAGGTGCAGCTGCA	Plasmid cloning
$p300_{BP}$-rev	ACGCTACTGAATTCCTTCTTTCCTAGTTCGTGCACTTTTCTTTA	Plasmid cloning
NLS-fwd	GTCTAGACGAATTCACGATCCAAAAAAGAAGAGAAAGG	Plasmid cloning
NLS-rev	TCAGCTAGGGTACCAGTACCTTTCTCTTCTTTTTTGG	Plasmid cloning
AF9-fwd	AGATTCGACTCGAGTAATGGCTAGCTCGTGTGCCGTGCAG	Plasmid cloning
AF9-rev	TACGACTTGGTACCGAGGATGTTCCAGATGTTTCCAGGTAACTC	Plasmid cloning
ENL-fwd	TCCGACTACTCGAGTAATGGACAATCAGTGCACCGTCCAGG	Plasmid cloning
ENL-rev	TACGACTTGGTACCGATGTGGCCACGGCCTCCAGGCA	Plasmid cloning
YEATS-rev	CGACATGGTACCGACACCCCGCCGGCCCGCAG	Plasmid cloning
ENL_C-fwd	CGACTACTCGAGTAATGGTAATGCCCGAAGGAGC	Plasmid cloning
ALKBH4-fwd	ACTACTGGGGATCCATGGCGGCGGCTGCCGCCGA	Plasmid cloning
ALKBH4-FLAG-rev	ATGTAGACGATATCTCATTTATCGTCATCGTCTTTGTAGTCGAACACGGGTCTTCCCTGGAAGGA	Plasmid cloning
ALKBH7-fwd	ACTACTGGGGATCCATGGCCGGGACTGGGCTGCTG	Plasmid cloning
ALKBH7-FLAG-rev	ATGTAGACGATATCTCATTTATCGTCATCGTCTTTGTAGTCGAAGCAGGCTGGGGGCGGCT	Plasmid cloning
ALKBH4-qPCR-fwd	TTCGGGAATGCGGTTG	qPCR
ALKBH4-qPCR-rev	CCTCGATCAGCATCACTC	qPCR
Beta-actin-fwd	CGTGGGGCGCCCCAGGCACCA	qPCR
Beta-actin-rev	TTGGCCTTGGGGTTCAGGGGGG	qPCR
